# Supplementary material for: Systematic Analysis of Genetic and Pathway Determinants of Eribulin Sensitivity across 100 Human Cancer Cell Lines from the Cancer Cell Line Encyclopedia (CCLE)
Source: Cancers (Basel). 2022 Sep 19;14(18):4532. doi: 10.3390/cancers14184532 (PMC9496846; doi:10.3390/cancers14184532)
Supplement: Supplementary file 1 [file cancers-14-04532-s001.zip › cancers-1888957-supplementary.pdf]

SUPPLEMENTAL FIGURES and TABLES

SUPPLEMENTAL FIGURES

Supplemental Figure S1

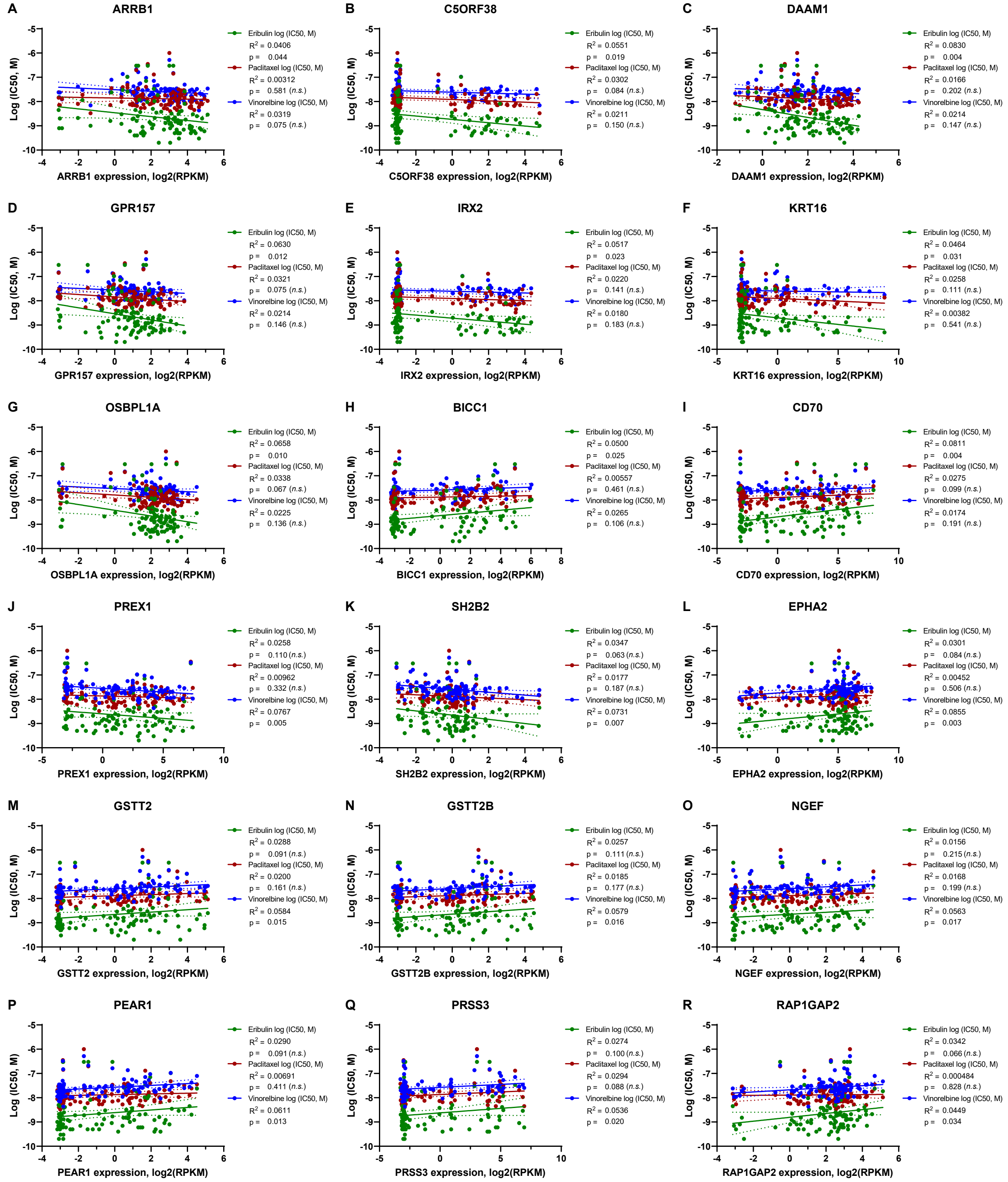

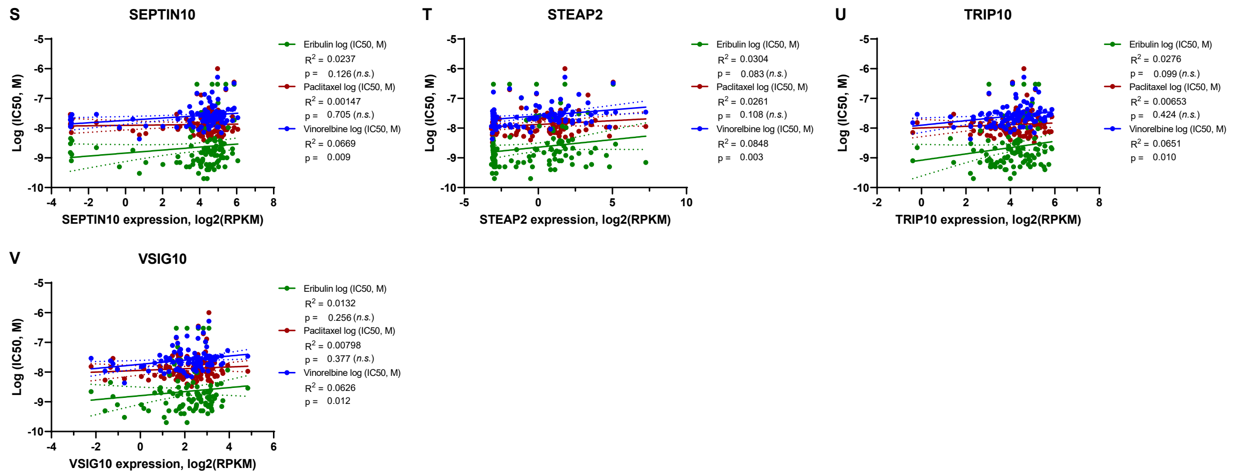

Supplemental Figure S1. Linear regression analyses of gene expression (log_2[RPKM]) versus log[M] IC50 for eribulin and vinorelbine UFGs. (A-G), eribulin up genes; (H, I), eribulin down genes; (J, K), vinorelbine up genes; (L-V), vinorelbine down genes. Linear regression lines (solid) are shown together with their corresponding 95% confidence bands (dotted).

Supplemental Figure S2

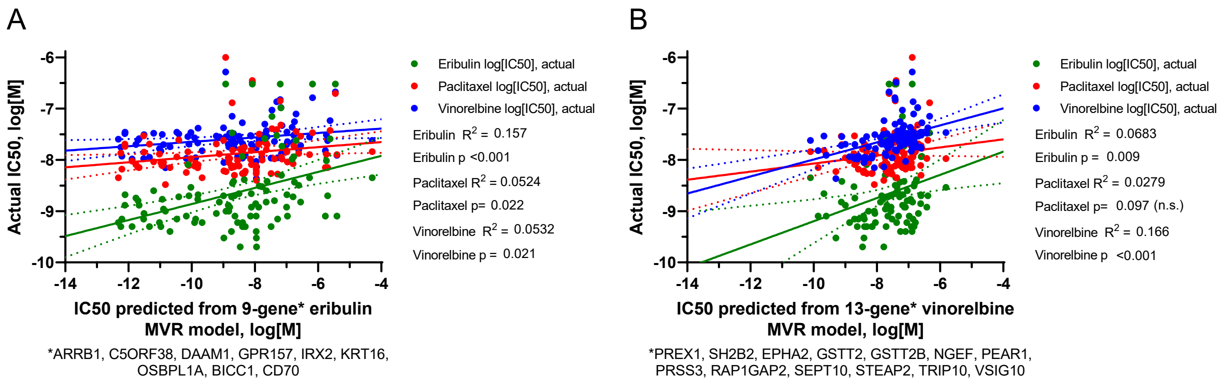

Supplemental Figure S2. Predicted versus actual IC50s based on full UFG set MVR models. Predicted IC50s were derived from (A) all 9 eribulin UGFs, and (B) all 13 vinorelbine UFGs. Linear regression lines (solid) are shown together with their corresponding 95% confidence bands (dotted). For visual comparison, scales of x- and y-axes were kept the same between both panels here and Panels A and C of Figure 5.

SUPPLEMENTAL TABLES

Supplemental Table S1: 100 CCLE cell lines and their antiproliferative IC50s against eribulin, paclitaxel and vinorelbine

Cell line	Tissue of origin	IC50, nM			Cell line	Tissue of origin	IC50, nM		
		Eribulin	Paclitaxel	Vinorelbine			Eribulin	Paclitaxel	Vinorelbine
769-P	Kidney	301[1]	33.9	163.7	LoVo	Colorectum	4.1	17.5	17.9
786-O	Kidney	301[1]	197.1	212.4	Malme-3M	Lung	0.2	16.2	23.8
8305C	H&N[2]-thyroid	3.1	31.8	25.1	MCF7	Breast	1.8	8.1	20
A2058	Skin	0.2	4	7.3	MES-SA	Uterus	2.5	15.8	36.9
A253	H&N[2]-salivary	1.6	7.2	24.4	MeWo	Skin	2	7.8	20.3
A2780	Ovary	1.9	8.4	21.6	MIA PaCa-2	Pancreas	0.6	14.6	20.6
A-375	Skin	5	11.3	13.2	MKN45	Gastric/esophagus	0.6	4.5	15.1
A498	Kidney	25.7	37.5	58.4	NALM-6	Blood	1.3	11	20.2
A549	Lung	3.7	18.1	55.8	NB4	Blood	0.3	5.9	4.3
A-673[3]	ST[4]/muscle	0.5	5.4	10.8	NCI-H1373	Lung	33.7	130	70.9
AN3 CA	Uterus	0.8	10.3	29.8	NCI-H1650	Lung	1.3	7.1	26
AsPC-1	Pancreas	2.9	10.6	34.2	NCI-H322	Lung	1.9	24.6	32.1
AU565	Breast	0.7	6.6	11.8	NCI-H441	Lung	0.6	13	16.6
BT-20	Breast	1.3	20.4	32.6	NCI-H446	Lung	0.3	6.1	8.9
BT-474	Breast	0.7	29.5	10.6	NCI-H460	Lung	1.9	7.1	23.4
BT-549	Breast	2	7.1	23.7	NCI-H508	Colorectum	11.7	16.8	53.2
BxPC-3	Pancreas	0.6	7.6	26.9	NCI-H522	Lung	1	8.9	17.9
CADO-ES1	Bone	301[1]	353.6	325.6	NCI-H716	Colorectum	301[1]	142.4	149.6
Caki-1	Kidney	9.6	12.9	30.5	NCI-N87	Gastric/esophagus	0.5	8.8	14.8
Caki-2	Kidney	3.2	13.4	15.7	NOMO-1	Blood	3	13.2	11.3
CAL-51	Breast	2.1	14.4	35	OE19	Gastric/esophagus	0.6	4.5	25.4
Calu-1	Lung	2	24.2	42.2	OVCAR-4	Ovary	2.2	7.6	28.9
Calu-3	Lung	29.9	39.1	136.3	OVCAR-8	Ovary	18.5	10.4	46.9
Calu-6	Lung	0.5	6	12.2	PANC-1	Pancreas	0.8	7.5	21.5
CAMA-1	Breast	0.9	14.1	31.2	PC-3	Prostate	0.9	20.5	29.2
Caov-3	Ovary	1.4	7.8	17.8	Pfeiffer	Blood	0.8	12.7	12.3
Capan-1	Pancreas	1.2	10.7	33.1	PLC/PRF/5	Liver	1.5	21.3	27.3
Capan-2	Pancreas	1.5	13.1	28.1	Raji	Blood	4.5	14.5	24
Daudi	Blood	0.8	7.1	15.2	RT4	Bladder	3.8	7.3	32.6
DU 145	Prostate	1.6	10.5	22.9	SK-BR-3	Breast	0.5	9.5	10.4
ES-2	Ovary	23.9	35.7	78.8	SK-HEP-1	Liver	4.5	12.7	28
FaDu	H&N[2]-pharynx	2.1	8	41.7	SK-LMS-1	Vulva	11.5	18.9	30.3
HCC1187	Breast	0.7	8.7	11	SK-MEL-28	Skin	0.5	5.5	12.1
HCC1428	Breast	0.5	20.3	11.3	SK-MEL-5	Skin	0.8	5.7	10.7
HCC-15	Lung	1	5.9	28.3	SK-MES-1	Lung	3.5	15.6	28.4
HCC1806	Breast	0.4	7.1	18.1	SK-OV-3	Ovary	1.2	20.3	21.4
HCC1937	Breast	0.5	13.6	17.6	SK-UT-1	Uterus	1.2	8.1	20.1
HCT 116	Colorectum	2	5	17	SW1353	Bone	3	9.9	30.4
HCT-15	Colorectum	301[1]	1,001[5]	518.2	SW48	Colorectum	0.4	7.7	18.5
HEC-1-A	Uterus	0.8	9.1	22.6	SW480	Colorectum	2.1	18.3	34.8
HEC-1-B	Uterus	0.7	5.3	11	T24	Bladder	103.4	48	105.5
Hep G2	Liver	26.2	21.9	49.9	T47D	Breast	2.5	9.2	33
HL-60	Blood	2.2	15.3	29.1	THP-1	Blood	3.8	19.8	28.6

Hs 578T	Breast	0.5	5.4	10.8	TOV-21G	Ovary	10.3	10.4	43.4
HT-1080	ST[4]/fibroblasts	0.4	6.6	9.3	U-2 OS	Bone	70.2	40.5	93.8
HT-29	Colorectum	1.7	7	21.5	U-87 MG	Brain	1.2	15	19.7
K-562	Blood	1.5	29	19.1	U-937	Blood	0.6	9.7	11.5
KP4	Pancreas	0.8	5.4	17	UACC-812	Breast	1.7	7.7	31.4
KYSE-150	Gastric/esophagus	1	3.3	7	ZR-75-1	Breast	1.1	9	19.9
LNCaP[6]	Prostate	0.7	11.4	34.6	ZR-75-30	Breast	8.1	14.4	28.5

Supplemental Table S1 footnotes:

[1] Eribulin IC50s for 5 cell lines (769P, 786-O, CADO-ES1, HCT15, NCI-H716) exceeded 300 nM, the top eribulin concentration tested. Therefore, IC50s of 301 nM for eribulin were assigned for these 5 cell lines.

[2] H&N: head & neck cancer with tissue of origin as identified.

[3] A-673 cell line was originally identified as rhabdomyosarcoma, but molecular cytogenetic characterization revealed it to be Ewing's Sarcoma (https://doi.org/10.1016/s0165-4608(03)00209-7). Muscle as tissue of origin remains correct.

[4] ST: soft tissue.

[5] Paclitaxel IC50 for HCT-15 cell line exceeded 1,000 nM, the top paclitaxel concentration tested. Therefore, the paclitaxel IC50 was assigned as 1,001 nM for HCT-15 cells.

[6] LNCaP clone FGC.

Supplemental Table 2. Genes positively and negatively associated with responses to eribulin, paclitaxel and vinorelbine at p < 0.0025 stringency[1]

Eribulin (n = 61)				Paclitaxel (n = 30)				Vinorelbine (n = 90)			
Gene	Up/ down	Fold-change[2]	p value	Gene	Up/ down	Fold-change[2]	p value	Gene	Up/ down	Fold-change[2]	p value
MAGEA3	Up	10.41285	0.00134	PPARGC1B[4]	Up	2.95803	0.00011	PREX1	Up	6.68057	0.00246
MAGEA2B	Up	6.38014	0.00213	ANKRD13B	Up	1.99038	0.00225	ST6GAL1	Up	5.38768	0.00080
CSAG1	Up	6.17522	0.00241	NCOA7	Up	1.91750	0.00066	TIAM1	Up	3.72193	0.00155
KRT16	Up	5.99757	0.00156	SLC35G1	Up	1.75572	0.00151	FAM46C	Up	3.35909	0.00142
NOTCH3	Up	5.24506	0.00238	SREBF1	Up	1.75556	0.00247	IRX5	Up	3.18898	0.00216
TRIB2	Up	4.56321	0.00117	SCO1	Up	1.60907	0.00016	SH2B2	Up	2.74440	0.00098
LYPD6B	Up	4.53307	0.00085	PDCD11	Up	1.56304	0.00142	MYLIP[3]	Up	2.70301	0.00003
B4GALNT3	Up	4.40125	0.00241	GID4	Up	1.54735	0.00055	PPARGC1B[4]	Up	2.68743	0.00030
IRX2	Up	4.36570	0.00202	COX10	Up	1.54379	0.00025	SH3BP5	Up	2.32253	0.00162
C5ORF38	Up	4.10552	0.00133	RAI1	Up	1.54306	0.00238	ST3GAL4[3]	Up	2.26131	0.00120
CD82	Up	3.29160	0.00214	RIOK1	Up	1.53980	0.00078	TNK2-AS1	Up	2.15335	0.00181
ARRB1	Up	2.90224	0.00030	TOP3A	Up	1.51873	0.00019	PDCD4	Up	2.11284	0.00158
KAZN	Up	2.78359	0.00136	C1QBP	Up	1.49609	0.00218	NBPF2P	Up	2.07988	0.00135
ADSSL1	Up	2.77511	0.00100	NEURL4	Up	1.49144	0.00023	KLHL24[3]	Up	2.01032	0.00133
DBP	Up	2.71818	0.00160	ZNF18	Up	1.46126	0.00188	TRIM62	Up	1.97540	0.00112
LY6E	Up	2.63749	0.00191	METTL16	Up	1.46050	0.00049	JARID2	Up	1.90211	0.00182
OSBPL1A	Up	2.43903	0.00210	MYBBP1A	Up	1.44647	0.00216	SMAP2	Up	1.85346	0.00044
GPR157	Up	2.34839	0.00165	POLR2A	Up	1.43184	0.00192	MYO9B	Up	1.79954	0.00005
MYLIP[3]	Up	2.23753	0.00060	DHX33	Up	1.42273	0.00185	CTU1[3]	Up	1.70662	0.00027
ST3GAL4[3]	Up	2.20709	0.00107	AKAP10	Up	1.39159	0.00105	ZNF316	Up	1.68257	0.00145
KLHL24[3]	Up	2.14127	0.00016	TNKS2	Up	1.36832	0.00118	RELT	Up	1.67809	0.00091
DAAM1	Up	2.13846	0.00133	PELP1	Up	1.35760	0.00160	IRF2BP1	Up	1.64703	0.00048
TNK2	Up	2.07354	0.00024	CLGN	Down	0.20938	0.00014	RCC2[3]	Up	1.63862	0.00025
CHKA	Up	1.80613	0.00156	BEX5	Down	0.31781	0.00222	ENTPD1	Up	1.63520	0.00222
CBX4	Up	1.80410	0.00074	BEND7	Down	0.32171	0.00112	ZNF324B	Up	1.63466	0.00167
ERCC6	Up	1.69932	0.00113	NRIP1	Down	0.34062	0.00210	LYPLA2P1	Up	1.62049	0.00133
MCCC1	Up	1.65605	0.00096	SUSD1	Down	0.43313	0.00190	DOT1L	Up	1.59021	0.00195
SUV420H2	Up	1.65596	0.00169	MYL6	Down	0.62106	0.00055	LIG1	Up	1.58071	0.00169
RCC2[3]	Up	1.64415	0.00038	MCFD2	Down	0.62650	0.00092	DDI2	Up	1.57856	0.00075
INAFM1	Up	1.63668	0.00100	SNF8	Down	0.65228	0.00046	CSK	Up	1.56816	0.00149
BRWD1	Up	1.59865	0.00092					ZBTB48	Up	1.53591	0.00201
CTU1[3]	Up	1.58029	0.00056					FARSA	Up	1.52700	0.00085
VPS13D	Up	1.54855	0.00035					FBXO46	Up	1.51772	0.00202
CC2D1A	Up	1.47589	0.00081					DENND4B	Up	1.51148	0.00086
RANBP9	Up	1.45607	0.00127					MYPOP	Up	1.51067	0.00180
ACAP2	Up	1.44057	0.00114					KMT2B	Up	1.49452	0.00040
DVL3	Up	1.43862	0.00107					ELL	Up	1.49166	0.00125
YEATS2-AS1	Up	1.43188	0.00183					TRMT2A	Up	1.47309	0.00066
AKAP8L[3]	Up	1.37659	0.00070					MAU2	Up	1.47040	0.00072
OPA1	Up	1.33365	0.00243					DDX49	Up	1.45256	0.00045
CD70	Down	0.11046	0.00056					DCPS	Up	1.45039	0.00234
BCAT1	Down	0.15199	0.00129					CHERP	Up	1.44976	0.00132
PAPSS2	Down	0.16298	0.00041					PPP6R1	Up	1.43986	0.00039
BICC1	Down	0.19316	0.00242					CLASRP	Up	1.43224	0.00022
PLAGL1[3]	Down	0.23053	0.00072					ZBTB17	Up	1.42310	0.00060
GLIS3	Down	0.26087	0.00096					BRD4	Up	1.41577	0.00144
PRKAA2	Down	0.28228	0.00128					CDK11A	Up	1.41249	0.00015
SPDL1	Down	0.56734	0.00165					C19ORF47	Up	1.40535	0.00115
CCNG1	Down	0.61505	0.00248					AKAP8L[3]	Up	1.39218	0.00181
RPL26L1	Down	0.64151	0.00176					CRTC2	Up	1.38189	0.00202
BAX	Down	0.64194	0.00120					ZNF574	Up	1.34107	0.00231
MRPL23	Down	0.64885	0.00072					UpF1	Up	1.32462	0.00207
TM2D2[3]	Down	0.65367	0.00150					SUpT5H	Up	1.32111	0.00028
NSA2	Down	0.67086	0.00080					CPSF3L	Up	1.30310	0.00176

ARPC3	Down	0.70837	0.00217					CDK11B	Up	1.27667	0.00166
EIF3F	Down	0.71221	0.00095					CXCL5	Down	0.07873	0.00044
SYS1[3]	Down	0.72230	0.00247					EREG	Down	0.10330	0.00068
PWP1	Down	0.72585	0.00150					TFPI	Down	0.11182	0.00063
SLC25A3	Down	0.72615	0.00171					WBP5	Down	0.13172	0.00023
WBP4	Down	0.72869	0.00191					HKDC1	Down	0.14089	0.00179
TMEM18	Down	0.75115	0.00167					DMTN	Down	0.14112	0.00005
								MYEOV	Down	0.14965	0.00096
								PRSS3	Down	0.16110	0.00236
								PEAR1	Down	0.17729	0.00005
								GSTT2	Down	0.19311	0.00011
								EPHA2	Down	0.19348	0.00131
								STEAP2	Down	0.20830	0.00215
								PLAGL1[3]	Down	0.21890	0.00143
								NGEF	Down	0.21979	0.00060
								GSTT2B	Down	0.22069	0.00012
								MARC2	Down	0.26060	0.00200
								LARP6	Down	0.28767	0.00223
								SEPTIN10	Down	0.29663	0.00183
								PDGFA	Down	0.30467	0.00220
								RAP1GAP2	Down	0.35885	0.00191
								ADAM9	Down	0.38507	0.00026
								LRRC49	Down	0.40109	0.00069
								TRIP10	Down	0.47549	0.00029
								VSIG10	Down	0.47800	0.00099
								TAX1BP3	Down	0.51789	0.00116
								TM2D2[3]	Down	0.52379	0.00001
								SPA17	Down	0.54261	0.00199
								TLDC1	Down	0.55524	0.00189
								HSDL2	Down	0.63273	0.00114
								AP2B1	Down	0.64461	0.00144
								CGRRF1	Down	0.65046	0.00083
								SYS1[3]	Down	0.65473	0.00059
								BOD1	Down	0.69145	0.00030
								CASC4	Down	0.71204	0.00162
								CYP20A1	Down	0.71937	0.00191

Supplemental Table 2 footnotes:

1. Positive and negative associations with response reflect higher and lower expression levels of specific genes between the most sensitive and least sensitive cell line quartiles, respectively.
2. Fold-changes represent ratios of mean expression levels of individual genes between most sensitive and least sensitive cell line quartiles.
3. The indicated 9 genes were shared by eribulin and vinorelbine. No genes were shared between eribulin and paclitaxel.
4. Only one gene was shared by paclitaxel and vinorelbine.

Supplemental Table 3: 100-gene networks identified by network propagation from eribulin and vinorelbine UFG sets							
Eribulin				Vinorelbine			
Gene[1]	Network Enrichment Score	Original UFG[2]	Shared[3]	Gene[1]	Network Enrichment Score	Original UFG[2]	Shared[3]
BICC1	4.42064	Yes		VSIG10	2.41402	Yes	
IRX2	4.32646	Yes		GSTT2	2.39356	Yes	
DAAM1	3.85316	Yes		PEAR1	2.37939	Yes	
OSBPL1A	3.83710	Yes		RAP1GAP2	2.36900	Yes	
KRT16	3.25369	Yes		STEAP2	2.36125	Yes	
CD70	2.71230	Yes		GSTT2B	2.34978	Yes	
ARRB1	2.05881	Yes		PREX1	2.27950	Yes	
SLC15A2	0.07794			PRSS3	2.24421	Yes	
RANBP6	0.05975			SH2B2	2.19373	Yes	
UBR2	0.05797			NGEF	2.12363	Yes	
B3GALT4	0.05622			SEPTIN10	2.00180	Yes	
UBR1	0.05550			TRIP10	1.81447	Yes	
DVL1	0.05483			EPHA2	1.41570	Yes	
PIFO	0.05482			GSTP1	0.06740		
SERHL2	0.05475			SHC1	0.04757		
BAALC	0.05419			THEM4	0.04383		
TFG	0.05415			ZPLD1	0.04337		
DMD	0.05380			ZAP70	0.03793		
OSBPL2	0.05324			TFPI	0.03533		
AMBP	0.05237			TRIM25	0.03469		Yes
POLG2	0.05209			SH2B1	0.03466		
FBXL16	0.05147			BZW1	0.03407		
SLC37A3	0.05110			CARD9	0.03393		
RHOD	0.05093			SYK	0.03364		
BSPRY	0.05075			SLC12A8	0.03364		
FBXL14	0.05073			SIRT2	0.03356		
MAPK3	0.05066			HNRNPL	0.03340		Yes
CTNNA3	0.04927			EPOR	0.03312		
CEP152	0.04923			DPEP1	0.03295		
BIN3	0.04819			XPO1	0.03276		
PEA15	0.04783			SORBS1	0.03215		
AHSA1	0.04769			C7ORF25	0.03161		
RHOC	0.04662			STAT3	0.03160		
C9ORF41	0.04653			ERBB2	0.03151		
TSC22D2	0.04558			CBL	0.03125		
CAPZA2	0.04525			NMD3	0.03096		
TMEM185A	0.04494			SH2D1A	0.03086		
ZNF331	0.04441			KIT	0.03066		
GNAZ	0.04352			RICTOR	0.03065		
CENPF	0.04352			SEPT14	0.03059		
EEA1	0.04316			SEPT12	0.03047		
ARMC6	0.04261			ELAVL1	0.03036		
KIFAP3	0.04221			AKT1	0.03011		
RAB5B	0.04097			SEPT4	0.02995		
GPN3	0.04027			SEPT1	0.02980		
PCNA	0.04018			MRS2	0.02958		
HEXIM1	0.03969			NTRK1	0.02957		
TUBB	0.03909			SEPT3	0.02942		
TUBA4A	0.03879			ACTR10	0.02934		
BCL6	0.03762			CDC42EP4	0.02924		
MAP3K7	0.03717			EPHA4	0.02915		
VAPB	0.03662			SEPT8	0.02905		
AURKB	0.03606			FAM118B	0.02898		
MTNR1B	0.03576		Yes	PDGFRB	0.02896		
PRPF40A	0.03560			ITSN1	0.02877		
PRPF31	0.03538			INSR	0.02873		

RHOA	0.03518			PPP1R14B	0.02859		
NPM1	0.03504			ESR2	0.02847		Yes
EP300	0.03503			JAK2	0.02840		
KRT82	0.03499			GRB2	0.02827		
CHD2	0.03460			TAF1D	0.02823		
PIK3R2	0.03447			MYC	0.02805		
IRF4	0.03367			SEPT5	0.02798		
VAPA	0.03358			INPPL1	0.02792		
INVS	0.03342			HCK	0.02785		
YY1	0.03307			MTOR	0.02768		
HIF1AN	0.03281			MRPL44	0.02753		
ZEB1	0.03279			LINC01587	0.02750		
ESR2	0.03254		Yes	C8ORF33	0.02750		
PTPRN2	0.03246			ACTR1B	0.02740		
HNRNPL	0.03245		Yes	SEPT6	0.02731		
TRIM25	0.03222		Yes	SEPT11	0.02703		
BCL3	0.03206			CTCF	0.02699		
KRT10	0.03181			SPDL1	0.02698		
POU2F2	0.03176			RPTOR	0.02650		
BCL11A	0.03171			YWHAZ	0.02650		
SMC3	0.03155			MTNR1B	0.02640		Yes
MEF2A	0.03137			PSMB5	0.02639		
BATF	0.03134			PRKCD	0.02636		
SP1	0.03101			RC3H1	0.02627		
PAX5	0.03098			YWHAE	0.02608		
NFKB1	0.03091			RPS6KB2	0.02598		
NFYA	0.03064			WDR83	0.02594		
RAD21	0.03059			LRCH3	0.02585		
EBF1	0.03040			SEPT2	0.02580		
CFTR	0.03036			SEPT7	0.02564		
TBP	0.03021			PRKCI	0.02554		
KRT5	0.03005			PSME4	0.02533		
SPI1	0.03003			HDGF	0.02527		
CDH1	0.03000			ZFP36L2	0.02521		
TCF12	0.02991			TFAP4	0.02513		
TNIP2	0.02941			TGS1	0.02505		
EFTUD2	0.02884			ALB	0.02501		
CUL3	0.02872			RGS3	0.02498		
HNRNPM	0.02871			DPF2	0.02489		
EGFR	0.02857			NEDD1	0.02476		
MAP2K3	0.02844			HK2	0.02469		
HNRNPA1	0.02835			SEPT9	0.02450		
NPHP1	0.02832			YTHDC1	0.02407		
UBASH3B	0.02825			PPP1CA	0.02406		

Supplemental Table 3 footnotes:

[1] Listed in order of decreasing network enrichment score.

[2] 7/9 eribulin UFGs and 13/13 vinorelbine UFGs were captured in the network propagation analyses. The 2 uncaptured eribulin UFGs were C5ORF38 and GPR157.

[3] Shared between eribulin and vinorelbine.
